# Characteristics and outcomes of patients triaged as critically ill in the emergency department of a tertiary care hospital in Bhutan

**DOI:** 10.1186/s12245-022-00468-8

**Published:** 2022-11-21

**Authors:** Sweta Giri, Melanie Watts, Shankar LeVine, Ugyen Tshering

**Affiliations:** 1 Khesar Gyalpo University of Medical Sciences of Bhutan, Thimphu, Bhutan; 2 Dartmouth Geisel School of Medicine, Hanover, USA; 3 Jigme Dorji Wangchuck National Referral Hospital, Thimphu, Bhutan

**Keywords:** Bhutan, Critically ill, Emergency department, Outcomes

## Abstract

**Background:**

In Bhutan, where the Emergency Medical System is forming and evolving, the number of acutely ill patients requiring critical care, both in the emergency departments and intensive care units, is steadily increasing. Given the lack of baseline data and the ever-increasing number of critical care patients, this study was aimed at describing the characteristics and outcomes of patients triaged as critically ill in the emergency department.

**Methods:**

An observational study was conducted over a yearlong period in the emergency department where all patients triaged as critically ill were approached for inclusion in the study. A case record form was used for the purpose of data collection. Epidata analysis was used for descriptive analysis and SPSS was used for binary logistic regression.

**Results:**

A total of 657 critically ill patients of all age groups visited the emergency department over the 1-year study period, with adults constituting the majority (81%). The majority (67%) of these patients had a favorable outcome of surviving to discharge. The most common diagnosis among critically ill neonates was neonatal sepsis. Among the critically ill pediatrics and adults, sepsis, respiratory illnesses, and trauma were the most common diagnoses. Intubation followed by mechanical ventilation and blood product transfusion were the most common lifesaving interventions performed on critically ill patients.

**Conclusion:**

The findings from this study constitute the first ever local database, at the national referral hospital in Bhutan, of critically ill patients treated in the emergency department. It highlights the central role the emergency department plays in their management and provides information for strengthening critical care services. It also highlights the areas of improvement and identifies high yield areas of training for the emergency department.

**Supplementary Information:**

The online version contains supplementary material available at 10.1186/s12245-022-00468-8.

## Background

Globally, there are an increasing number of patients, including the critically ill, being treated in emergency departments (EDs) [1]. Critical illness is defined as any immediate, life-threatening, reversible condition, which, if left untreated, results in poor outcome or death [2]. Management of the critically ill starts with resuscitation and is followed by close titration of therapy according to the evolution of the disease process—this usually starts in the ED and continues in intensive care units (ICUs) [1].

Critically ill patients get recognized upon arrival to EDs via various screening methods. At the tertiary center where this study was conducted, this identification is facilitated by the World Health Organization-International Committee of the Red Cross (WHO-ICRC) integrated triaging tool (Additional file 1). The WHO-ICRC triaging tool categorizes patients into red, yellow, or green criteria based on their presenting symptoms, signs, and vital signs. Once the severity of illness/injury is determined, patients are disposed to the appropriate treatment areas: resuscitation bay, standard ED bed, or green zone. Patients triaged as red require time-sensitive evaluation and resuscitation, accounting for the vast majority of critically ill patients.

In Bhutan, where emergency and critical care medicine are currently being developed, there is a lack of data regarding the types of critical cases seen, interventions required, and patient outcomes. Given the lack of baseline data and the ever-increasing number of critical care patients in the ED, there is a need to study the current profile of critical care patients in the tertiary hospital, which serves as the nation’s apex health center.

To understand the relevance of a single-center observational study of all patients triaged as critically ill in Bhutan’s only tertiary care national referral center, it is necessary to have a basic understanding of the structure of Bhutan’s medical system: points of access to care, hospitals, and referral system. The national referral center has the country’s main adult ICU, only intensivist, only neonatal ICU, and pediatric ICU and houses the country’s only specialists in several areas including neurosurgery, cardiology, nephrology, and surgical and pediatric subspecialists. As a result, critical referrals come to this hospital from across the country contributing significantly to the volume and variety of critical patients triaged and cared for in the ED. Most referrals are transported via ground ambulances with the geographical distribution, mountainous terrain, and road conditions often contributing to long transport times. Among the critical referral cases, some who meet the criteria are transported by helicopter emergency medical services (HEMS). Given the limited distribution of resources in low- and middle-income countries (LMICs), we suspect that this structure has a number of similarities to other countries, particularly in the region.

This study was carried out with an aim of creating a database of the critically ill in the ED at Bhutan’s only national referral hospital. This will enable the department and the hospital to plan areas of priority for staff training and procurement of equipment and better understand the variety and resuscitative needs of critical patients. It will also help identify areas requiring didactic focus and procedural training, since the residency program in emergency medicine is in its budding stage. Findings from this study may be used as a guiding material for planning resource allocation and education/training needs for emergency services that are at a similar stage of development.

## Methods

### Study design

An observational study was conducted among patients triaged as critically ill in the ED of a tertiary care hospital, which serves as the national referral hospital in Bhutan. The study was conducted for a period of 1 year, beginning October 1, 2020, till September 30, 2021.

### Study setting and population

This study was conducted in the ED of a tertiary care hospital, which provides emergency treatment to patients from all age groups. The ED caters to the population of the region as well as patients who are referred from other hospitals across the country via ground or air ambulance, in its capacity as the national referral center.

All patients, irrespective of age, undergo triage on presentation to the ED. The WHO-ICRC integrated tool is used for triaging purposes, wherein patients meeting any of the red criteria or the presence of any of the high-risk vital signs are directly brought into the resuscitation bay/red zone and are evaluated immediately by a doctor. These patients are deemed critically ill by triage criteria.

There are four beds in the resuscitation bay, three for adults and one for pediatric/neonates, with a provision to expand capacity to six using extra trolleys if the situation demands. Resuscitation, stabilization, and treatment of critical patients occur in this area prior to being transferred to ICUs or downgraded to the yellow zone for transfer to the general wards. Patients meeting the yellow criteria upon initial triage are sent to the yellow zone, which has 22 beds. Patients in the yellow zone can be upgraded to the red zone/resuscitation bay if they clinically deteriorate.

ED staffing comprises 2 emergency physicians, 8 emergency medicine residents, 7 medical officers, and 35 nurses [as of September 2022]. At least one emergency physician is present in the department from 9 am to 9 pm every day and on-call from 9 pm to 9 am. A combination of emergency medicine residents and medical officers staff the department 24 h a day. Nurse to patient ratio varies between 1 nurse to 4–6 patients.

We included all patients of any age group, irrespective of being referred from other hospitals or self-presenting, who are triaged as critically ill by the triage nurse. We also included patients who are re-triaged and upgraded to being critically ill from other areas of the emergency department. We excluded those who are brought in dead and those who left against medical advice.

### Sampling and recruitment

In order to ensure that the study design is reproducible and it contributes to the existing pool of literature, this study proceeded as a census and included all patients triaged as critically ill by the triage nurse of the ED over the 1-year study period. A yearlong period was decided upon to ensure that all types of critical illness, including seasonal diseases resulting in critical illnesses, are also well represented. Patients in the red zone/resuscitation bay of the ED who met the inclusion criteria were approached for recruitment into the study. Informed written consent was obtained from all patients above the age of 18 years who had the capacity to provide informed consent. For those who were not able to provide informed consent and for patients under the age of 18 years, informed written consent was obtained from the primary caregiver while informed assent was obtained from the patient. Details on the informed assent form were explained to the patient in a language that they understood if they were unable to read or were too sick to read. Recruitment into the study had no impact on patient care.

### Data collection

Data collection was done using a case record form, which was constructed based on inputs from co-authors and was pilot tested among 10 potential participants before finalizing the working version.

### Data analysis

Data analysis was done using Epidata version 2.2.2.182 and SPSS Statistics 26. Continuous variables were descriptively reported as medians with minimum to maximum range while categorical variables were reported as frequencies and proportions. Association between patient characteristics [categorical variables] and outcomes [categorical variables] was tested using binary logistic regression. *P* values < 0.05 were considered statistically significant.

## Results

**Fig. 1:**
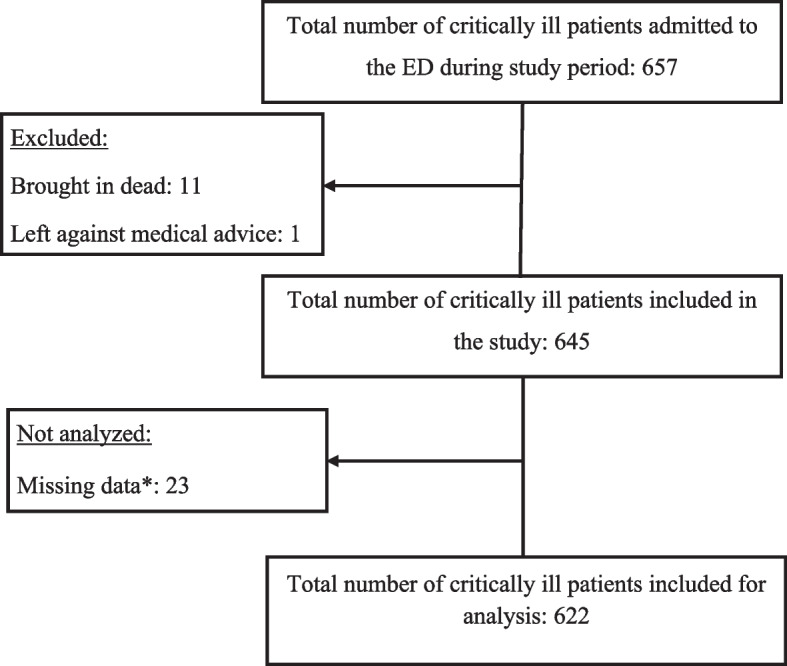
Flow chart showing the number of patients enrolled and analyzed in the study. *Missing data includes those with an absence of two or more triage vital signs

During the yearlong study period, 657 critically ill patients were admitted to the ED, which comprised 11.8% of all ED admissions. After excluding the 11 who were brought in dead, 1 who left against medical advice, and 23 with missing data, a total of 622 [94.7%] with complete information were included in the analysis, as illustrated in Fig. 1.

### Patient characteristics

Among the 622 critically ill patients analyzed in the study, adults comprised the majority [81.3%] with 54% being males. Patient characteristics are displayed in Table 1.

**Table 1:** Characteristics of critically ill patients

Characteristics	Neonates^**a **^***n*** (%)	Pediatrics^**b **^***n*** (%)	Adults^**c **^***n*** (%)
**Sex**			
Male	21 (53.8)	45 (58.4)	270 (53.3)
Female	18 (46.2)	32 (41.6)	236 (46.7)
**Co-morbidities**			
None	39 (100)	66 (85.7)	204 (40.3)
One	0	11 (14.3)	228 (45.1)
Two or more	0	0	74 (14.6)
**Manner of presentation**			
Referral	29 (74.3)	24 (31.2)	229 (45.3)
Self-presentation	10 (25.7)	53 (68.8)	277 (54.7)
**Total**	39	77	506

^a^Neonates: ≤ 28 days old; *n*=39

^b^Pediatrics: 29 days–12 years; *n*=77

^c^Adults: ≥12 years; *n*=506

The reason for the current classification of patients aged 29 days to 12 years as pediatrics is based on the hospital pediatric definition as this determines which department the patient is disposed to. Similarly, although those aged 12–18 years are not truly adults as per WHO definition, their management is done in the adult medical/surgical sub-specialty wards and adult ICUs. Thus, all patients above the age of 12 years have been categorized as adults

All critically ill patients presented with at least one abnormal vital sign, which was noted by the triage nurse during the triaging process. Nearly 50% of critically ill neonates had tachycardia and 44% were hypoxic. Among the critically ill pediatric population, nearly 65% each had altered sensorium and abnormal heart rates. Similarly, among the critically ill adults, nearly 70% had abnormal blood pressure recordings and nearly 50% each were hypoxic and had altered sensorium.

While in the ED, the critically ill patients required lifesaving interventions as part of initial resuscitation and further stabilization. Among all interventions, “intubation and mechanical ventilation” was the most performed intervention [26.8%, *n*=167] across all three subgroups of critically ill. Table 2 depicts the various interventions and their frequencies.

**Table 2:** Lifesaving interventions performed on the critically ill patients

Interventions	Neonates ***n*** (%)	Pediatrics ***n*** (%)	Adults ***n*** (%)
CPR	0 (0)	5 (6.5)	71 (14.0)
Cardioversion/defibrillation	0 (0)	0 (0)	18 (3.6)
Noninvasive ventilation	0 (0)	0 (0)	90 (17.8)
Intubation and mechanical ventilation	4 (10.2)	20 (26.0)	143 (28.3)
Tube thoracostomy	0 (0)	0 (0)	15 (3.0)
Central line insertion	0 (0)	0 (0)	105 (20.7)
Emergent hemodialysis	0 (0)	0 (0)	51 (10.1)
Blood product transfusion	0 (0)	2 (2.6)	84 (16.6)
Others^a^			4 (0.8)

^a^Others include thrombolysis and chemical pacing

**Fig. 2:**
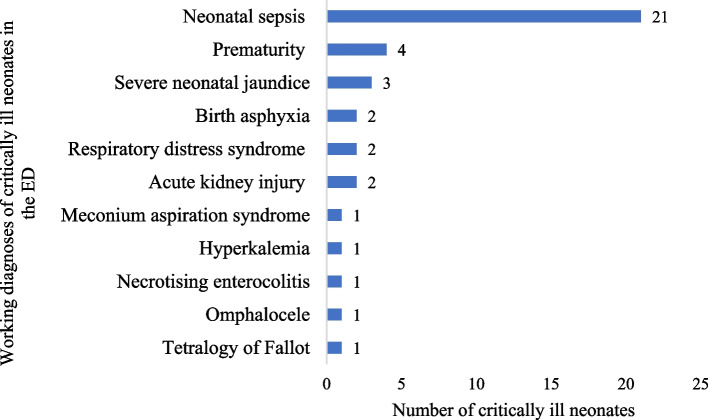
Working diagnosis of critically ill neonates admitted to the ED during the study period (*n*=39)

**Fig. 3:**
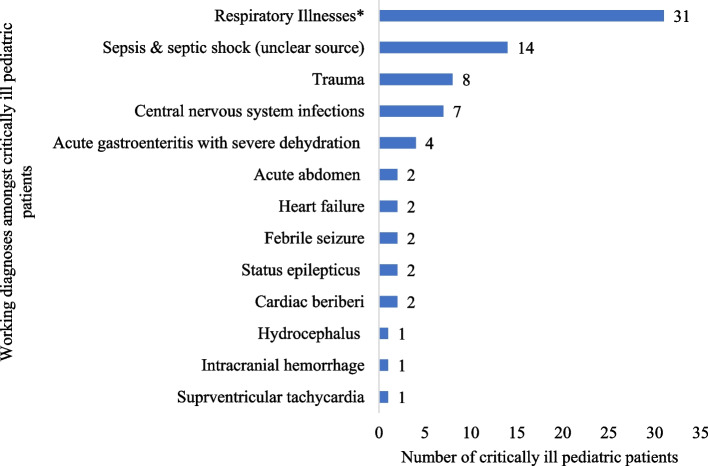
Working diagnosis of critically ill pediatric patients admitted to the ED during the study period (*n* = 77). *Respiratory illnesses include pneumonias, asthma exacerbations, bronchiolitis, croup, and acute respiratory distress syndrome

**Fig. 4:**
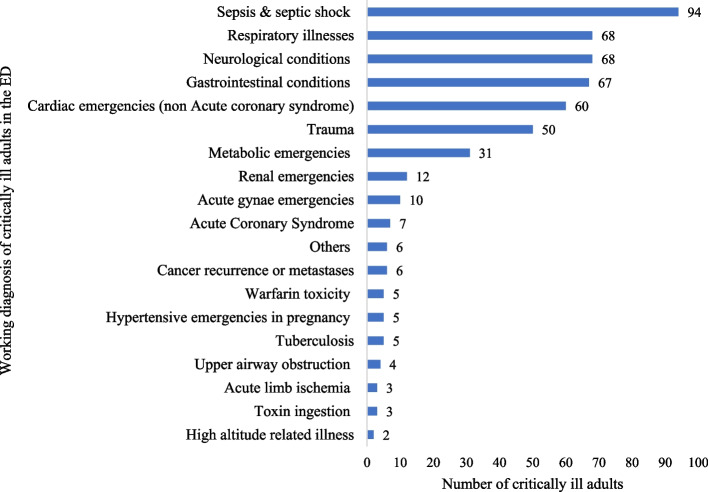
Working diagnosis of critically ill adults admitted to the ED during the study period (*n*=506). Details on diagnoses included within each of the diagnostic categories are listed in Additional file 2

The critically ill patients had a myriad of working diagnoses while they remained boarded at the ED, receiving resuscitative and stabilization measures. Among neonates, the most common diagnosis was neonatal sepsis [53.8%, *n*=21]. Respiratory illnesses [44.9%, *n*=31] were the most common diagnoses among the pediatric critically ill. Sepsis and septic shock [20.6%, *n*=94] were the most common diagnoses among critically ill adults. Trauma accounted for nearly 10% of critically ill patients in pediatrics and adults; there were no traumatic injuries in the neonatal age group. Figures 2, 3, and 4 provide detailed working diagnoses of critically ill patients on disposition from the ED.

The critically ill patients were admitted to ICUs and wards after they were stabilized. While all critically ill neonates were admitted to the neonatal ICU, the majority of pediatric [67.5%] and adult patients [40%] were admitted to the pediatric ICU and adult ICU. They remained boarding in the ED for varying amounts of time, subject to the availability of beds in the admitting units. The median ED length of stay [LOS] during the study period for these critically ill patients by age group was 1 h and 21 min for neonates, 2 h and 12 min for pediatrics, and 18 h and 11 min for adults.

### Patient outcomes

Patient outcomes are either survival to hospital discharge or no survival to hospital discharge/death. Survival to hospital discharge indicates that the patient survives till discharge, irrespective of the remnant deficits.

Nearly 67% [*n*=414] of all critically ill patients who presented to the ED survived to hospital discharge. The overall mortality rate was 33.4%, of which 36% [*n*=75] died in the ED itself. The pediatric subgroup of critically ill patients had the highest survival rate at 88.3% compared to the survival rates of the neonatal and adult groups which were 82% and 62% respectively (Table 3).

**Table 3:** Survival to discharge outcomes of the critically ill patients

Type of patient	Survived to discharge ***n*** (%)	Did not survive to discharge ***n*** (%)
Neonates	32 (82.0)	7 (18.0)
Pediatrics	68 (88.3)	9 (11.7)
Adults	314 (62.0)	192 (38.0)

Characteristics common to all three subgroups of critically ill patients were tested for associations with the outcome. This included the presence of underlying co-morbidities and critical lifesaving interventions that were performed in at least 2 of the 3 subgroups. Patients with no underlying co-morbidities had higher odds of surviving to discharge by more than two folds [OR: 2.4; 95% CI: 1.685–3.354]. The absence of requiring cardiopulmonary resuscitation, intubation, and blood product transfusion was significantly associated with survival to discharge outcome [*P* < 0.001]; in other words, those who did not require these interventions had higher odds of surviving to discharge (Table 4).

**Table 4:** Predictors of survival to discharge

Characteristics	Survived to discharge	Did not survive to discharge	Crude OR	***P*** value	aOR	***P*** value
**Presence of co-morbidities**						
No	235 (76.0)	74 (24.0)	Ref	0.000	2.71 (1.72–4.27)	0.000
Yes	179 (57.2)	134 (42.8)	2.37			
**Interventions**						
**Cardiopulmonary resuscitation**						
No	406 (74.4)	140 (25.6)	Ref	0.000	9.25 (4.01–21.30)	0.000
Yes	8 (10.5)	68 (89.5)	24.65			
**Intubation and mechanical ventilation**						
No	358 (78.7)	97 (21.3)	Ref	0.000	5.23 (3.22–8.51)	0.000
Yes	56 (33.5)	111 (66.5)	7.31			
**Blood transfusion**						
No	372 (69.4)	164 (30.6)	Ref	0.000	1.70 (0.96–3.01)	0.000
Yes	42 (48.8)	44 (51.2)	2.37			

The association between ED LOS and survival to discharge outcomes was assessed using a logistic regression model. For the purpose of logistic regression, the two subgroups of neonates and pediatrics have been grouped together as “non – adult patients” as illustrated in Table 5.

**Table 5:** Comparison of outcomes based on ED length of stay of the critically ill patients admitted to the ED during the study period (*n*=622)

LOS	Survived to discharge ***n*** (%)	Did not survive to discharge ***n*** (%)	Crude OR (95% CI)	***P*** value
**Non-adults**				
ED LOS ≤6 h	88 (85.4)	15 (14.6)	0.48 (0.05–4.04)	0.507
ED LOS > 6 h	12 (92.3)	1 (7.7)		
**Adults**				
ED LOS ≤6 h	69 (69.7)	30 (30.3)	1.52 (0.94–2.44)	0.082
ED LOS> 6 h	245 (60.2)	162 (39.8)		

Irrespective of the ED LOS among both adults and non-adults, there was no significant association (*P*>0.005) with outcomes, in terms of survival or death.

## Discussion

Among the 622 patients triaged as critically ill during the yearlong study period at this national referral hospital, 45.3% were referred from district hospitals which by age grouping comprised nearly 3/4th of the neonates, 1/3rd of the pediatrics, and nearly half of adult patients in the study. Overall, 54.7% of the patients in the study self-presented from the local catchment area. Among all the patients, males slightly outnumbered females with a male to female ratio of 1.17:1. The majority of the patients in this study required at least one critical lifesaving intervention in the ED among which the most common intervention performed was intubation and subsequent mechanical ventilation [26.8%, *n*=167]. Sepsis and trauma were the most common working diagnoses [20.8% and 10% respectively]. Nearly 67% of all critically ill patients survived to hospital discharge.

Much of the burden of infectious disease and sepsis is in LMICs [3]. In our study, sepsis and septic shock was the most common diagnosis among neonates and adults, accounting for 53.8% and 20.6% respectively of all diagnoses; in the pediatric population, it accounted for 18.2% of all diagnoses, second only to respiratory illnesses. It can be challenging to compare the prevalence of sepsis in studies across the region given that diagnostic classifications in observational hospital studies are often by organ system which can be the septic source but is not necessarily separately classified as sepsis. A study done assessing acute care needs in Kerala, India, had similar results with tropical infections and sepsis accounting for a combined 20% of ED diagnoses [4].

In practice, LMICs are often reliant on research and treatment guidelines from the high-income countries, where the prevalence of illnesses and the availability of both diagnostic and treatment modalities differ. This study combined with other regional studies demonstrates the high prevalence of infectious diseases and it seems prudent to focus clinical care interventions and medical education in these areas. For instance, sensitization of triage nurses on the early detection of sepsis via vital signs and presenting symptoms could help improve the timely recognition of septic patients. This could be coupled with initiatives to enhance care of septic patients in our department such as a focus on the timely initiation of antibiotics and fluid resuscitation.

It is established that death and disability from trauma are profound in LMICs [5]. In this study, trauma accounted for nearly 10% of critically ill pediatric and adult patients which is similar to findings from studies in neighboring countries. Trauma comprised 8.4% of ED visits in a study from India and data from a Sri Lankan ED reports that trauma constituted the majority of all surgical cases seen in their ED [4, 6]. To ensure that a certain minimum level of care is available for every injured person worldwide, the World Health Organization published the *Guidelines for Essential Trauma Care* [7]. These guidelines provide a list of essential trauma care services required for various types of traumas. Since our center saw a substantial number of critically ill patients secondary to trauma, it would be worthwhile conducting a needs assessment of trauma care capabilities using the guidelines as a template. Such a study was conducted in Vietnam and it aided in the identification of several low-cost measures for strengthening trauma care for different levels of healthcare delivery centers [8]. As recommended by authors who profiled ED visits in Sri Lanka, having a dedicated in-house trauma team, particularly for critical traumas, would overcome the need to contact and call for the presence of individual team members, thus reducing the time from injury to critical interventions and surgery [6, 9].

ED boarding is common and increasing worldwide [10]. The median LOS for the critically ill neonates and pediatric population in our study was approximately 2 h. The ED LOS of critically ill adults in our study was 18 h; this is substantially higher than those of our neighboring countries, Nepal and Pakistan [11, 12]. A shortage of critical care beds in our hospital which has only one adult ICU with 10 beds that must care for all sub-specialties is a likely contributor to the long critical adult ED LOS. The situation is similar in Nepal where the ED has started to function as an extension of the ICU. The authors, however, advocate for increasing ICU bed strength because EDs are not designed nor staffed to function like ICUs [11]. That low-resource settings see a disproportionate amount of the global burden of critical illness and the need for a higher proportion of hospital beds in these settings to be allocated as critical care beds has been demonstrated in prior studies [2]. The adult ED LOS data from our study supports these findings.

In our study, nearly 67% of all critically ill patients survived to discharge with an overall mortality rate of 33.4%. Of this, 36% died in the ED itself and critically ill adults had the highest mortality rate at 38%. The mortality rate in our ED is higher than those reported by studies conducted in the region [4, 11]. The prolonged ED LOS among critical adult patients likely contributed to this high ED mortality rate as other studies in the region reported significantly shorter ED LOSs [6, 11, 12]. Our study also included critically ill patients whose goals of care included DNR/DNI and comfort care where all attempts were made to keep the patient comfortable but not necessarily to prolong life.

It is hard to know the impact that the COVID pandemic may have had. Although the study was planned before the first COVID case in Bhutan, the timing of the study coincided with the peak of the COVID pandemic and lockdowns in the country. This necessitated resources and staff to be stretched staffing the ED, COVID hospitals, flu clinics, and holding areas. This resulted in higher nurse to patient ratios than usual, which could have contributed to the higher mortality. Lockdowns and patients’ concerns around coming to the hospital during the pandemic may have resulted in delayed presentation of patients to the hospital, contributing to increased morbidity and mortality. Finally, as previously mentioned, the tertiary center where this study was conducted receives referrals of the sickest of patients from across the country often having to overcome long transportation times which could have also contributed to the higher mortality rate. Ongoing tracking of this data or repeating a similar study in the future could help us better understand the degree of contribution of these various factors to ED mortality.

## Conclusion

This yearlong study identified clinical characteristics and outcomes of patients triaged as critically ill in the emergency department of the national referral hospital in Bhutan. The distribution of diagnoses among different age groups and most common procedures provides helpful data to identify high yield areas for didactic focus and procedural training. The high proportion of critical sepsis and trauma patients suggests that potential future intervention aimed at early detection, timely assessment by appropriate clinical teams, and early initiation of treatment among these patients may be prudent. There is a need to better understand the mortality figures through ongoing data collection or future-focused study. The need to increase the capacity of ICU beds and improve critical care capacity with better staffing and targeted trainings is identified.

### Limitations

One of the biggest limitations of this study is that critically ill patients were not classified into subgroups by the widely used APACHE II scores as has been done in similar studies elsewhere. This score could not be used because components of the score include parameters such as pH. Due to a limited supply of blood gas cartridges, it is not possible to calculate parameters like pH in all of our critically ill patients. The difficulties around the use of the APACHE score and similar prognostic models for critically ill patients in areas where resources are limited have been previously studied in South Asia [13].

Patient outcomes have been limited to survival to discharge and non-survival to discharge. Total hospital length of stay or neurologic deficits at the time of discharge has not been calculated or presented.

A number of the months of the study period coincided with the national lockdowns due to COVID which would have affected the data in numerous ways including delays to hospital presentation due to patient concerns around exposures and challenges with transportation. Furthermore, the pandemic negatively affected staffing patterns and may have led to delayed time to critical interventions due to the mandatory need for COVID testing. These factors likely had varied effects on the volume and timing of critical patient presentations and may have led to differences in mortality rates. Although the COVID mortality in Bhutan was low, it is worth mentioning that COVID patients were triaged to a COVID-specialized temporary hospital and therefore are not included in this study.

## Supplementary Information

**Additional file 1.** The WHO-ICRC integrated triaging tool.

**Additional file 2.** Details of various diagnoses among critically ill adults under major categories.

## Data Availability

The datasets used and/or analyzed during the current study are available from the corresponding author on reasonable request.
