# Supplementary figures and images for: Characteristics and outcomes of patients triaged as critically ill in the emergency department of a tertiary care hospital in Bhutan
Source: Int J Emerg Med. 2022 Nov 21;15:64. doi: 10.1186/s12245-022-00468-8 (PMC9682814; doi:10.1186/s12245-022-00468-8)

**Additional file 1**


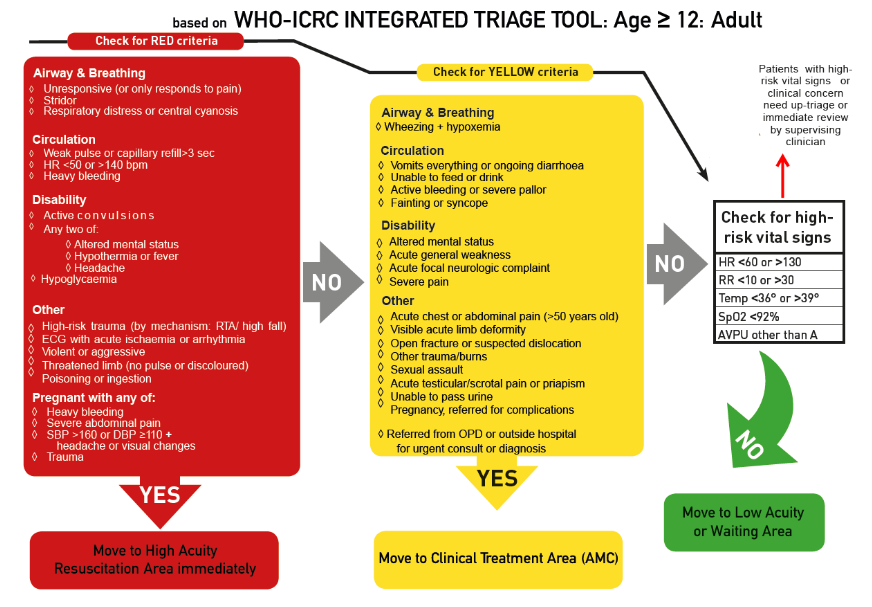

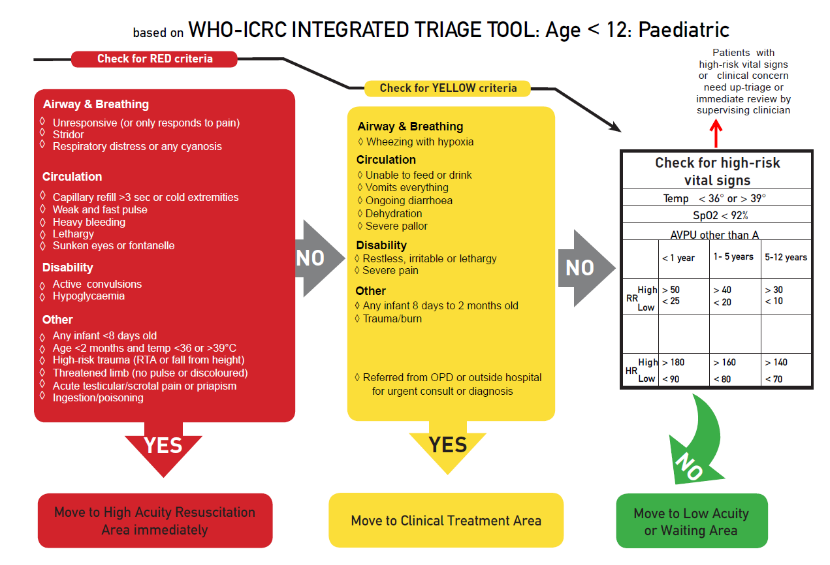

Supplement: Supplementary file 1 — Additional file 1. The WHO-ICRC integrated triaging tool. [file 12245_2022_468_MOESM1_ESM.docx]
