# Supplementary material for: Characteristics and outcomes of patients triaged as critically ill in the emergency department of a tertiary care hospital in Bhutan
Source: Int J Emerg Med. 2022 Nov 21;15:64. doi: 10.1186/s12245-022-00468-8 (PMC9682814; doi:10.1186/s12245-022-00468-8)
Supplement: Supplementary file 2 — Additional file 2. Details of various diagnoses among critically ill adults under major categories. [file 12245_2022_468_MOESM2_ESM.docx]

**Details of various diagnoses among critically ill adults under major categories**

*Sepsis & septic shock* includes: sepsis, neutropenic sepsis, septic shock and metabolic acidosis

*Respiratory illnesses* include: chronic obstructive pulmonary disease exacerbations, bronchial asthma exacerbations, interstitial lung disease exacerbation, bronchitis, type 2 respiratory failure, pneumonia, aspiration pneumonitis, adult respiratory distress syndrome, pulmonary embolism, post procedural respiratory complications and pleural effusion

*Neurological conditions* include: hemorrhagic stroke, ischemic stroke, cerebral venous thrombosis, status epilepticus, meningitis, encephalitis and brain abscess

*Gastrointestinal conditions* include: gastrointestinal bleeding, acute surgical abdomen (burst appendix, bowel perforation, mesenteric ischemia, bowel obstruction), acute pancreatitis, acute liver failure, Cholangitis, hepatic encephalopathy and hepatorenal syndrome

*Cardiac (non ACS) emergencies* includes: heart failure, cardiogenic shock, cardiac dysrhythmias, atrioventricular and bundle branch blocks, cardiomyopathies, pulmonary edema, acute and sub-acute endocarditis and aortic dissection

*Trauma* includes: traumatic brain injury (epidural hematoma, subdural hematoma, traumatic intracranial hematoma, traumatic subarachnoid hemorrhage, diffuse axonal injury, penetrating brain injury), blunt and penetrating chest trauma resulting in intra thoracic organ injury, blunt and penetrating abdominal trauma resulting in intra abdominal organ injury, pelvic fracture, extremity fracture, spinal injury, extensive soft tissue injury and polytrauma.

*Metabolic emergencies* include*:* diabetic ketoacidosis, hypoglycemia, hyperosmolar hyperglycemic syndrome, alcoholic ketoacidosis, thyroid storm

*Acute obstetrics & gynaecological emergencies* include: molar pregnancy, miscarriages, ectopic pregnancy, ruptured ectopic pregnancy, ante partum hemorrhage, pyelonephritis in pregnancy, pre eclampsia, eclampsia, pregnancy induced hypertension

*Renal emergencies* include: uremic encephalopathy, refractory hyperkalemia and pyelonephritis in pregnancy

*ACS (Acute Coronary Syndrome)* includes: STEMI, NSTEMI and Unstable angina

*Upper airway obstruction* includes: Ludwig’s angina, anaphylaxis

*High altitude related illnesses* include: High altitude cerebral edema (HACE), High altitude pulmonary edema (HAPE)

*Others* include: alcohol withdrawal syndromes, attempted suicide by hanging, electrocution and snake bite
